# Supplementary material for: Green Solvents in the Extraction of Bioactive Compounds from Dried Apple Cultivars
Source: Foods. 2023 Feb 19;12(4):893. doi: 10.3390/foods12040893 (PMC9957507; doi:10.3390/foods12040893)
Supplement: Supplementary file 1 [file foods-12-00893-s001.zip › foods-2209677-supplementary.pdf]

Supplementary material

Green Solvents in the Extraction of Bioactive Compounds from Dried Apple Cultivars

Marcela Hollá, Veronika Pilařová, František Švec, Hana Sklenářová

Department of Analytical Chemistry, Faculty of Pharmacy in Hradec Králové, Charles University, 50005 Hradec Králové, Czech Republic

Table S1. Physicochemical properties of selected bioactive compounds, including molecular weight, Log P, and pKa, obtained from Scifinder database, and retention time with selected wavelength used for detection from UHPLC-DAD method used for the analysis of all samples.

**Compound**	**Summary formula**	**Molecular weight**	**Log P**	**pKa**	**Structure**	**Retention time [min]**	**Selected λ [nm]**
caffeic acid	$C_9H_8O_4$	180.16	0.663±0.286	4.58±0.10	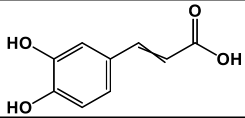	7.42	320
catechin	$C_{15}H_{14}O_6$	290.27	0.610±0.454	9.54±0.10	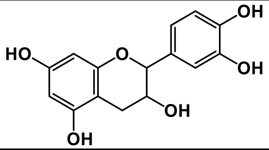	7.31	280
chlorogenic acid	$C_{16}H_{18}O_9$	354.31	0.370±0.461	3.91±0.50	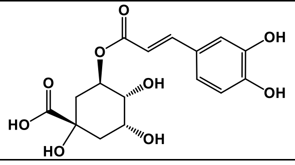	5.39	320
(-)-epicatechin	$C_{15}H_{14}O_6$	290.27	0.610±0.454	9.54±0.10	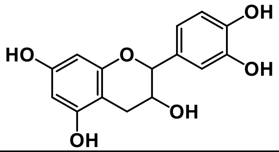	5.89	280
gallic acid	$C_7H_6O_5$	170.12	0.531±0.325	4.33±0.10	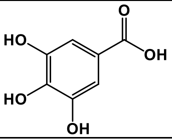	2.69	280
guaiaverin	$C_{20}H_{18}O_{11}$	434.35	0.043±1.357	6.17±0.40	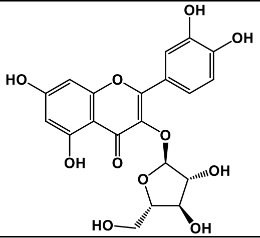	10.17	254
hirsutrin	$C_{21}H_{20}O_{12}$	464.38	0.111±1.370	6.17±0.40	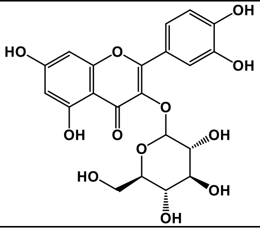	10.34	254
hyperoside	$C_{21}H_{20}O_{12}$	464.38	0.111±1.370	6.17±0.40	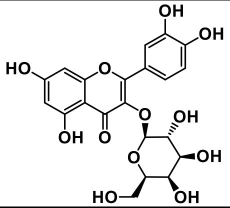	11.24	254
phloretin	$C_{15}H_{14}O_5$	274.27	2.514±0.384	7.16±0.40	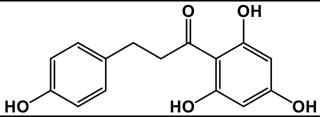	14.23	280

phloridzin	$C_{21}H_{24}O_{10}$	436.41	0.365±0.385	7.15±0.40	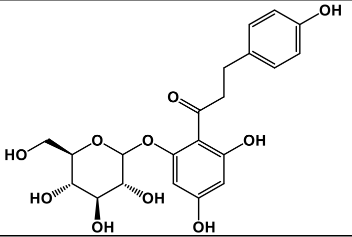	12.25	280
quercetin	$C_{15}H_{10}O_{7}$	302.24	1.989±1.075	6.31±0.40	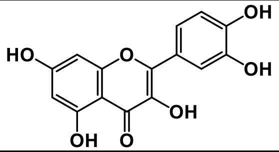	13.47	354
quercitrin	$C_{21}H_{20}O_{11}$	448.38	0.579±1.358	6.17±0.40	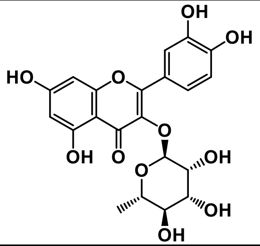	11.68	254
reynoutrin	$C_{20}H_{18}O_{11}$	434.35	0.043±1.357	6.17±0.40	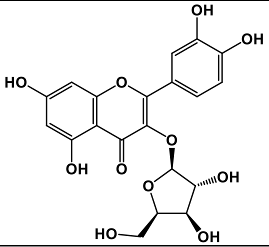	10.96	254
rutin	$C_{27}H_{30}O_{16}$	610.52	0.903±1.416	6.17±0.40	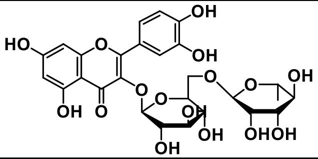	9.47	354

Table S2. Optimized GXLE conditions suggested by Optimizer for TPC in dried apples. N15 – optimal setpoint 1, O – optimal setpoint 2, R – robust point.

Experiment	CO_2 [vol. %]	H_2O in co-solvent [vol. %]	Temperature [°C]	Pressure [bar]	Probability of failure [%]	TPC [µg/g]
O1	31.4	19.8	46	197	4.8	703
O2	31.4	18.7	58	145	2.9	752
O3	21.7	12.7	41	292	4	701
O4	17.6	11.2	41	300	7.2	701
O5	36.7	19.4	50	164	3.3	828
O6	40.8	19.7	41	152	3.4	979
O7	16	18.5	35	280	8.7	1100
O8	40	19.6	39	295	5	758
O9	44.0	17.9	75	127	5.3	884
O10	33.5	19.8	45	174	3.5	1012
O11	16	9.5	75	280	4.9	1159
O12	32.6	8.9	80	283	4	1432
O13	34	18.5	75	120	5	1717
O14	52	18.5	75	120	3.9	1477
O15	16	18.5	75	280	28	1097
O16	46	19.25	78	290	6.7	1082
O17	34	16.25	58	210	3.1	514
O18	40	12.5	80	300	4.3	1022
O19	10	12.5	55	300	6.1	792
O20	40	20	80	200	15	1570
O	70	10	47	193	1	1003
R	46	11	50	180	0.1	830

TPC – total phenolic content in extract

Table S3. Optimized UE conditions suggested by Optimizer for TPC in dried apples. N15 – optimal setpoint 1; O – optimal setpoint 2; R – robust point.

**Experiment**	**Ethanol [vol. %]**	**Temperature [°C]**	**Probability of failure [%]**	**TPC [µg/g]**
O1	26.1	70	0	-
O2	25.7	70	0	-
O3	26.2	70	0	-
O4	26.1	70	0	-
O5	26.1	70	0	-
O6	26.1	70	0	-
O7	26.6	70	0	-
O8	26.7	70	0	-
O	26.7	70	0	2225 ± 25
R	26.7	61	0	2208 ± 39

TPC – total phenolic content in extract

Table S4: Comparison of developed GXLE and UE methods on results of different apple cultivars.

**Phenolic compound [µg/g]**	**Meteor**		**Golden delicious**		**Topaz**		**Artiga**		**Angold**	
	**GXLE**	**UE**	**GXLE**	**UE**	**GXLE**	**UE**	**GXLE**	**UE**	**GXLE**	**UE**
catechin	296	312	230	242	112	101	125	122	239	295
chlorogenic acid	155	177	279	366	225	221	546	560	740	1054
epicatechin	146	134	0	5	71	81	75	77	52	187
guaiaverin	126	113	76	64	258	227	53	44	31	29
hirsutrin	217	191	90	74	207	204	269	234	84	78
hyperosid	107	98	34	30	45	43	56	49	26	28
phloridzin	153	153	102	92	40	42	44	51	35	48
reynoutrin	47	44	17	16	42	33	81	72	43	43
rutin	20	20	1	1	10	11	65	72	10	14
**TPC (%RSD)**	**1296 (0.8)**	**1255 (2.1)**	**849 (3.7)**	**898 (0.4)**	**1033 (0.0)**	**975 (0.3)**	**1341 (1.7)**	**1294 (0.1)**	**1288 (2.5)**	**1785 (0.3)**

Table S5. Comparison of phenolics in different apple cultivars and paired t-test values.

Cultivar/ Analyte [µg/g]	Meteor		Golden Delicious		Topaz		Artiga		Angold		t value
	GXLE	UE	GXLE	UE	GXLE	UE	GXLE	UE	GXLE	UE	
catechin	296.3	311.6	230.0	241.7	111.5	100.9	124.6	122.2	239.2	295.2	0.92
chlorogenic acid	155.0	177.1	279.2	366.5	225.1	221.3	546.2	559.7	740.3	1054.4	1.40
epicatechin	145.6	134.4	0.0	4.8	71.3	80.9	75.3	77.0	52.5	187.3	2.27
guaiaverin*	126.2	112.6	76.0	64.5	257.6	227.1	52.8	44.1	31.5	29.5	2.15
hirsutrin*	217.4	190.8	89.5	74.4	206.8	203.8	268.9	234.0	84.1	77.9	2.07
hyperoside*	106.6	97.7	33.5	29.9	44.9	42.6	56.3	48.8	26.3	27.6	1.69
phloridzin*	153.0	152.9	101.6	91.8	40.2	41.7	43.7	50.8	35.2	47.9	0.60
quercetin	4.2	0.5	0.5	0.5	4.3	2.9	4.0	2.2	0.5	0.5	1.71
quercitrin*	10.2	8.8	5.3	2.9	4.6	5.1	10.2	6.7	1.4	3.5	0.63
reynoutrin*	47.1	44.1	16.5	16.4	41.7	33.3	81.1	72.2	42.5	42.7	1.72
rutin*	20.0	20.5	0.7	0.7	9.7	10.8	65.2	71.9	9.9	14.1	2.00

* glycosidic phenolic substances; t-critical value: 2.35

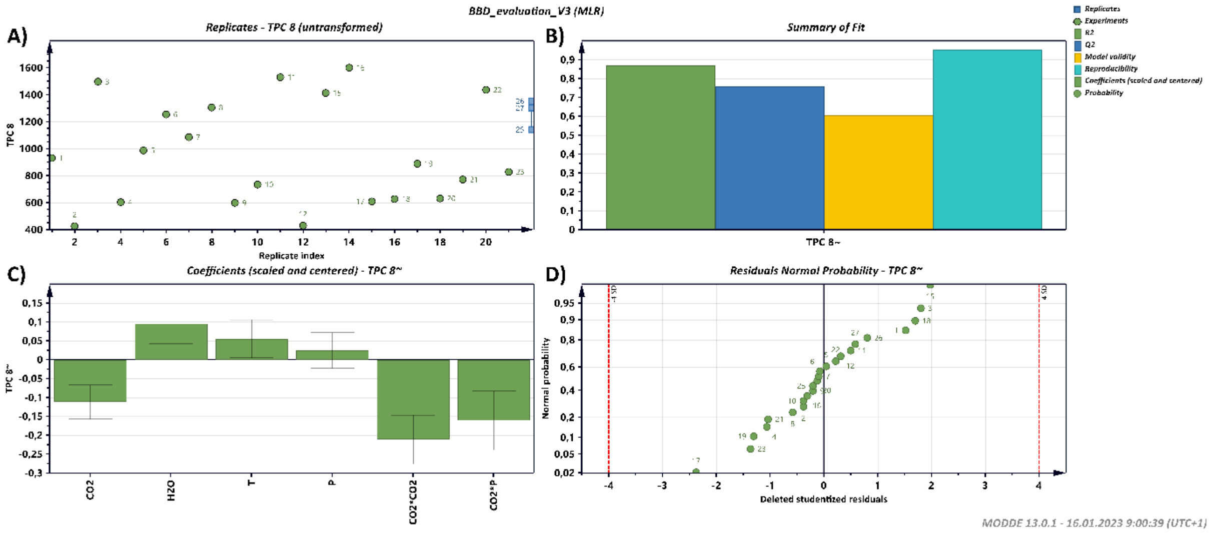

Figure S1. Model analysis of Design of experiments for GXLE with MODDE software. A) Replicate plot for Total Phenolic Content (TPC) determined as extracted amount with experimental numbers. The replicates are shown as blue squares. B) Summary of fit for the TPC extraction. R^2 – linearity, Q^2 – predictability. C) The coefficient box plot for single parameters and their interactions affecting the TPC. D) Residual plot for TPC. The red dash lines indicated fourfold standard deviation.

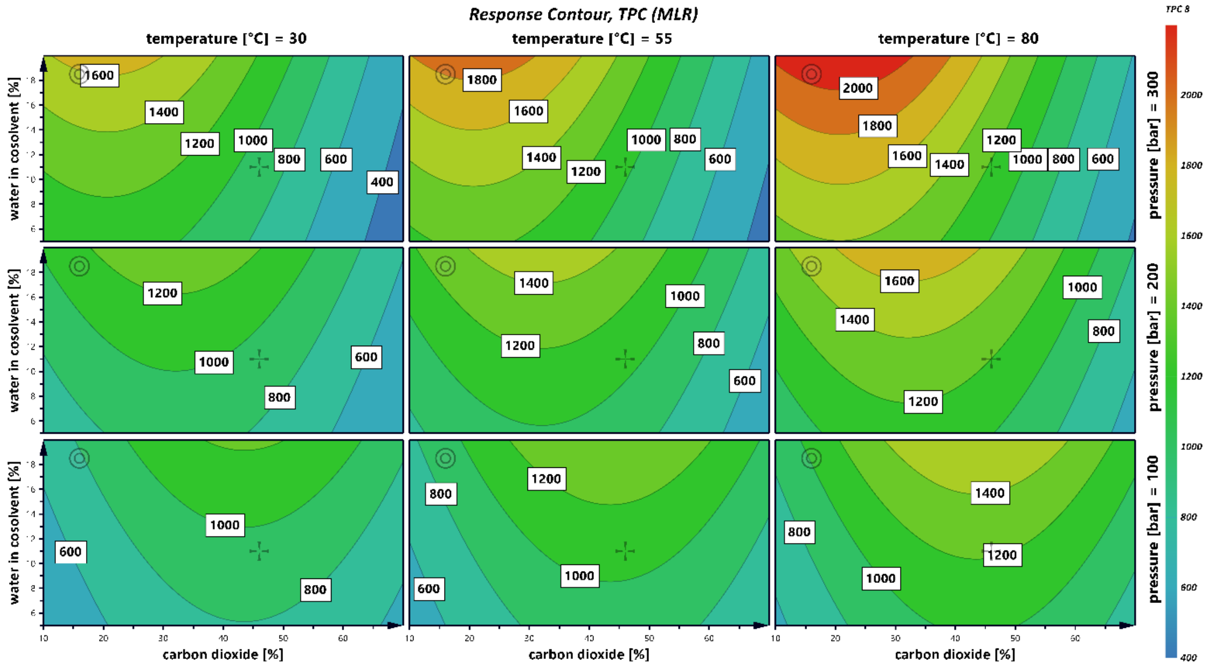

Figure S2. Contour response plot for TPC summarizing the effect of individual parameters optimized in GXLE.

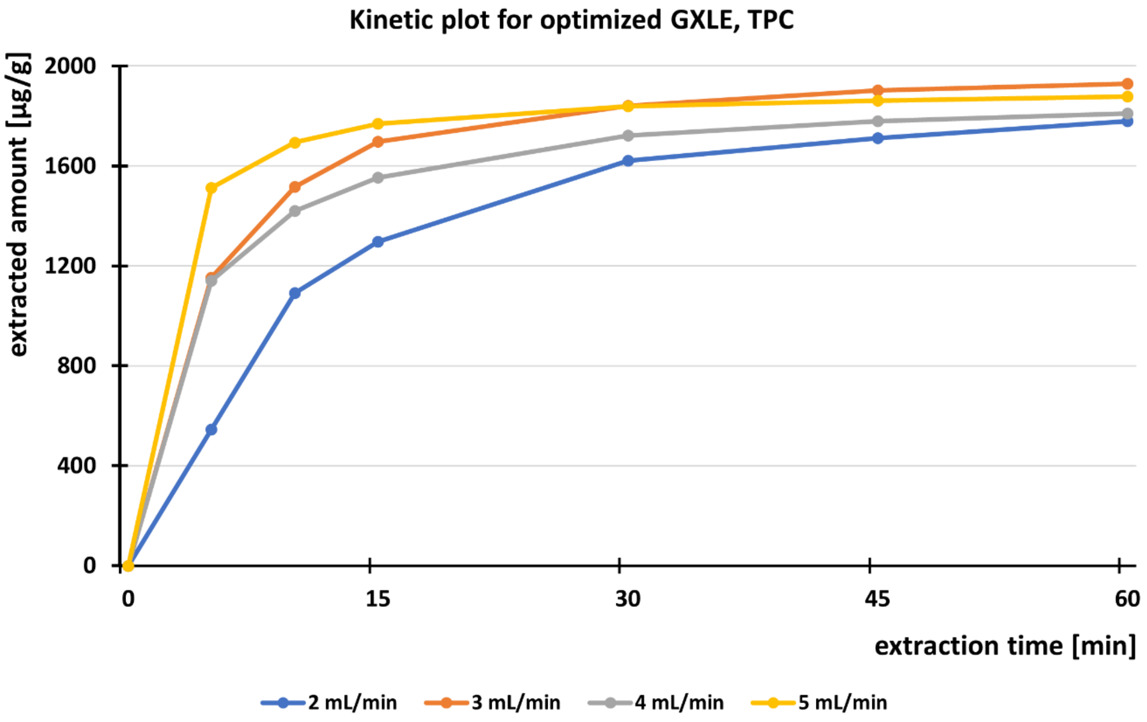

Figure S3. The extraction kinetic plot for the evaluation of the flow rate effect and optimization of the extraction time for GXLE carried out with CO_2/EtOH/H_2O (34/53.8/12.2; v/v/v) solvent, 70 °C and 120 bar.

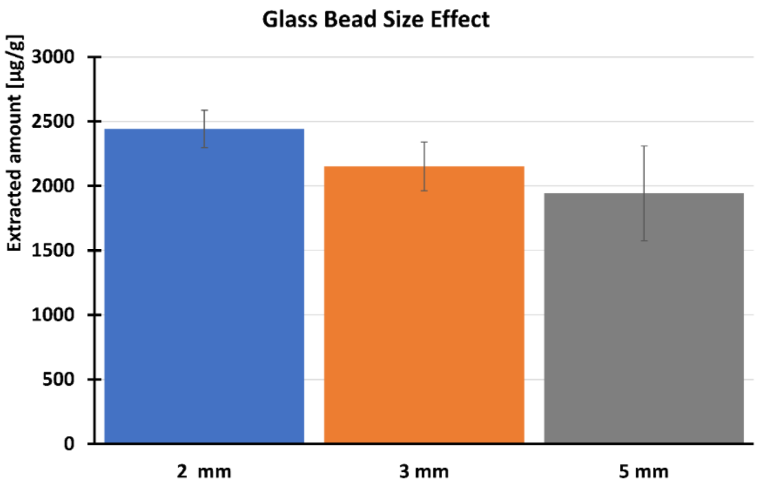

Figure S4. Effect of glass beads size used as a dispersant in sample extracted by optimized GXLE. Error bars show standard deviation calculated from 3 replicates. GXLE conditions: extraction solvent CO_2/EtOH/H_2O (34/53.8/12.2; v/v/v) solvent; temperature 70 °C; pressure 120 bar; flow rate 3 mL/min; time 30 min.

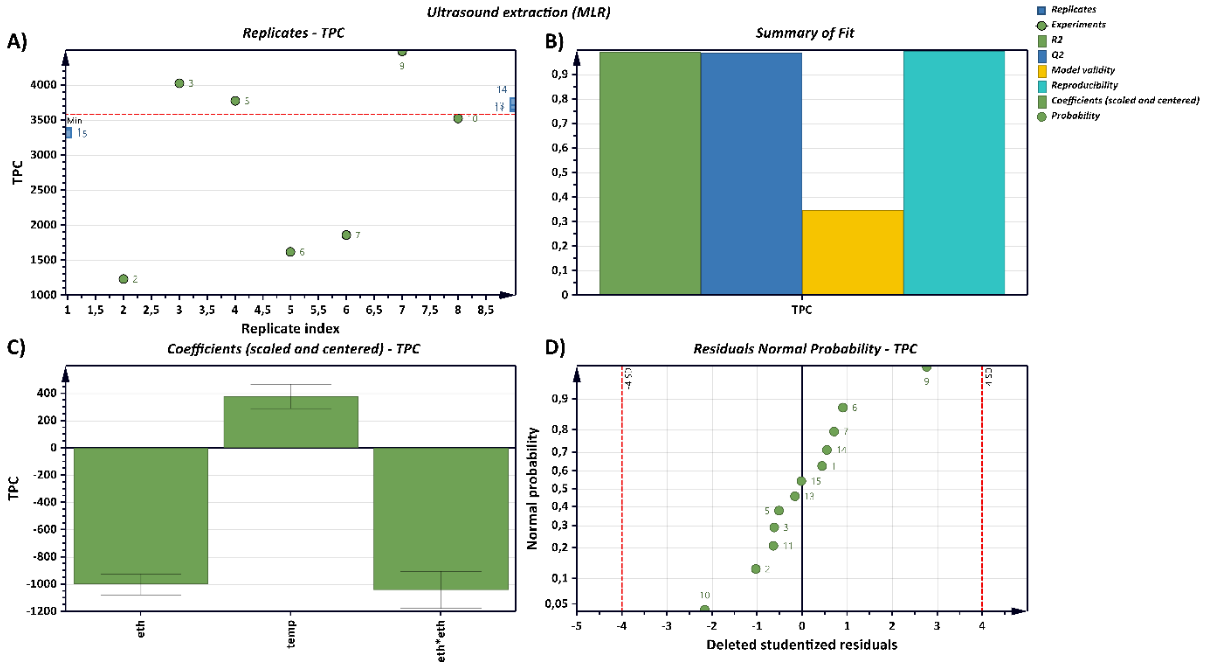

Figure S5. Model analysis of Design of experiments for UE with MODDE software. A) Replicate plot for TPC determined as extracted amount with experimental numbers. The replicates are shown as blue squares. B) Summary of fit for the TPC extraction. R^2 – linearity, Q^2 – predictability. C) The coefficient box plot for single parameters and their interactions affecting the TPC. D) Residual plot for TPC. The red dash lines indicated fourfold standard deviation.

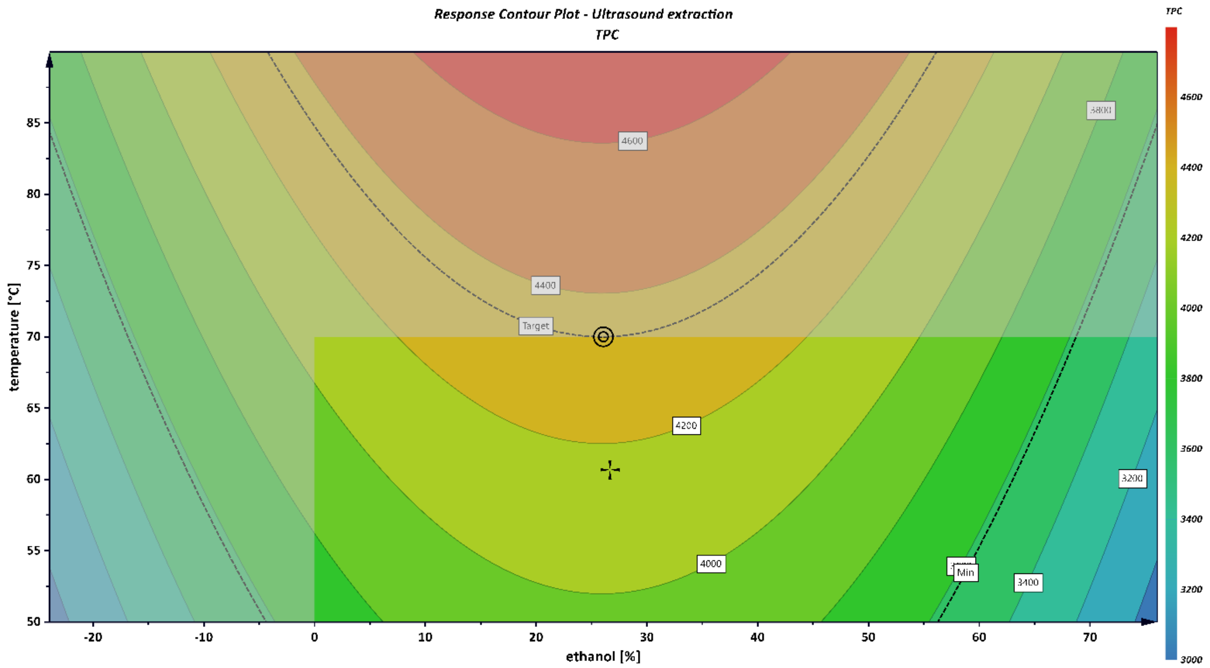

Figure S6. Contour response plot for TPC summarizing the effect of individual parameters optimized in UE.

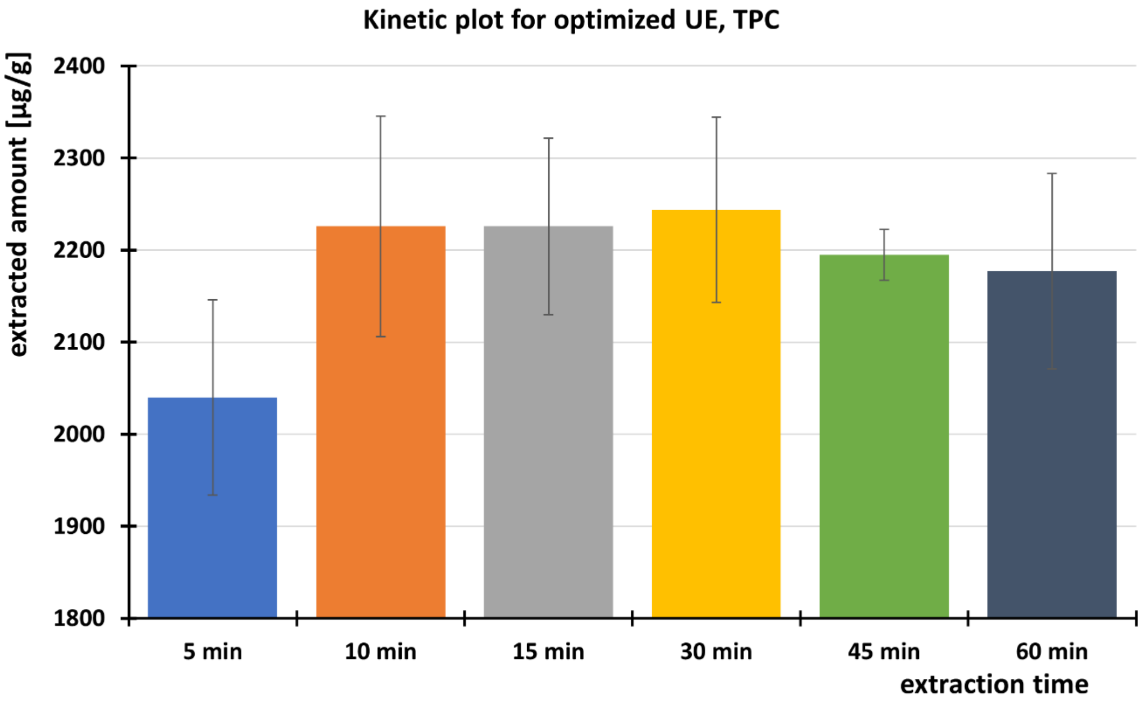

Figure S7. The extraction kinetic plot for the optimization of the extraction time for UE.

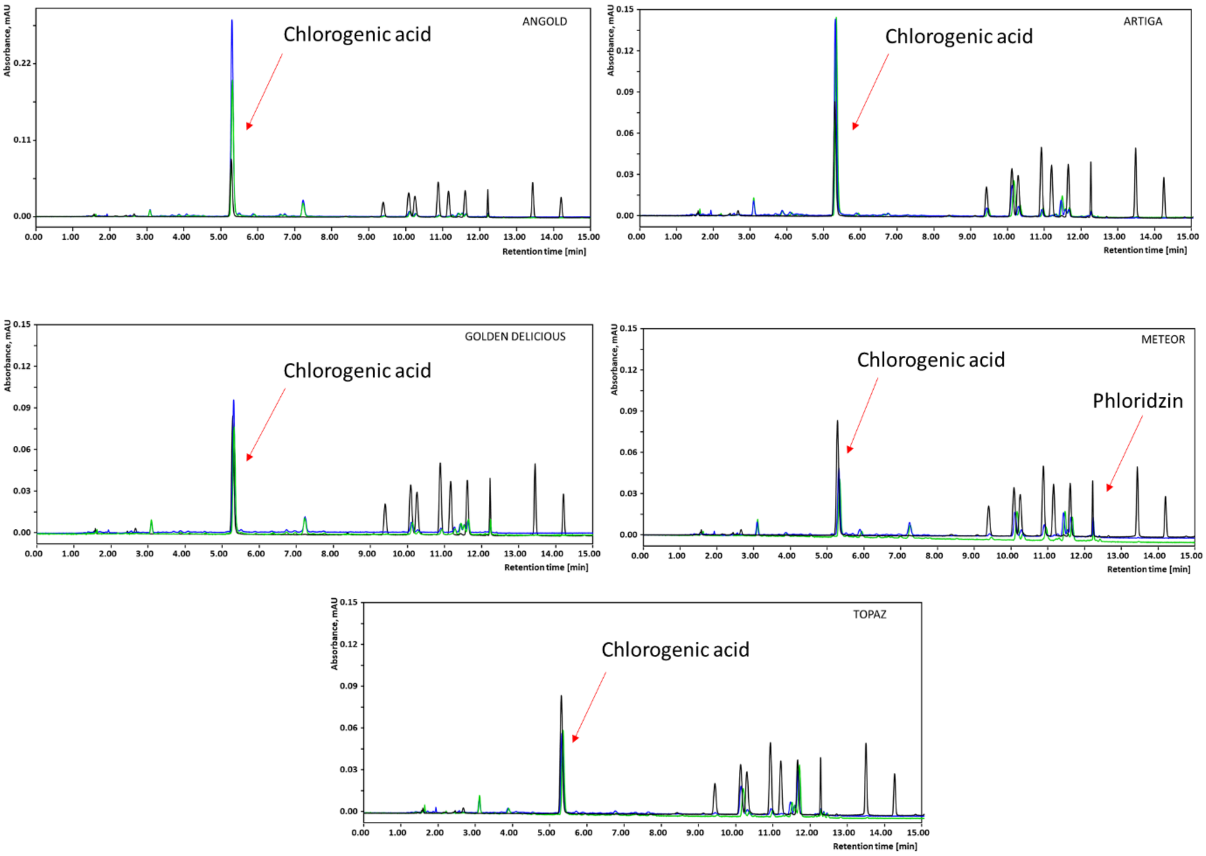

Figure S8. Chromatograms of phenolics separation – standard in black, GXLE in green, and UE in blue (all at 320 nm).

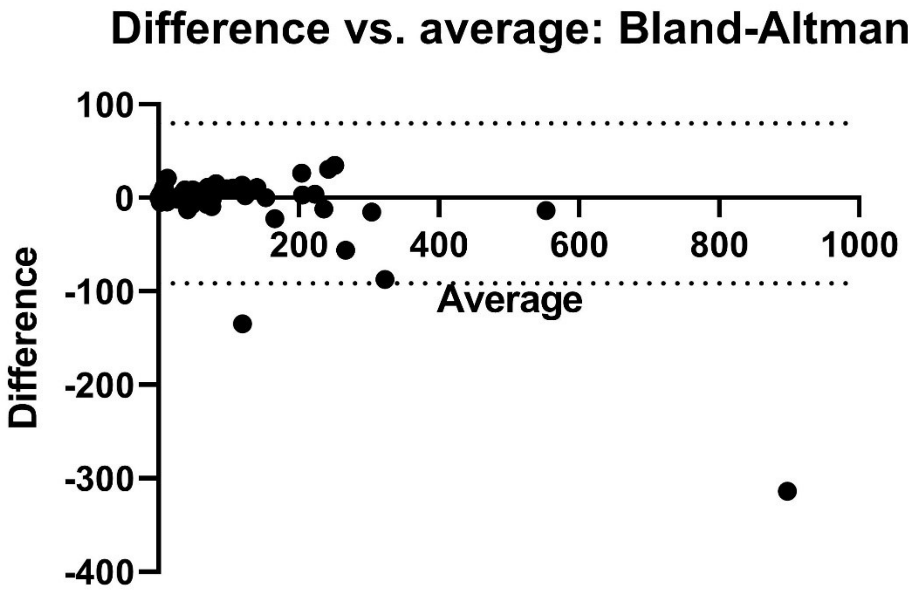

Figure S9. Bland-Altman test comparing the extracted amounts of phenolics using GXLE and UE. Two outliers were identified, for epicatechin and chlorogenic acid (variety ‘Angold’, UE). For other compounds, GXLE and UE provided comparable results.

Analytical Greenness report sheet

27/07/2022 14:46:57

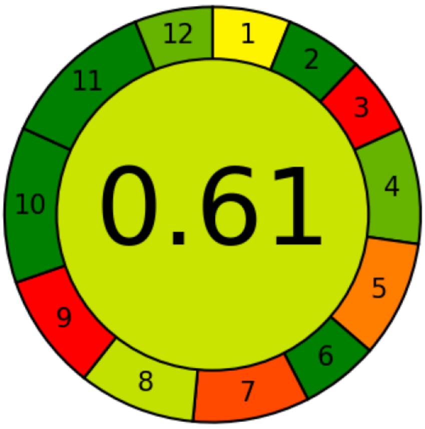

Criteria	Score	Weight
1. Direct analytical techniques should be applied to avoid sample treatment.	0.48	2
2. Minimal sample size and minimal number of samples are goals.	1.0	2
3. If possible, measurements should be performed in situ.	0.0	2
4. Integration of analytical processes and operations saves energy and reduces the use of reagents.	0.8	3
5. Automated and miniaturized methods should be selected.	0.25	3
6. Derivatization should be avoided.	1.0	2
7. Generation of a large volume of analytical waste should be avoided, and proper management of analytical waste should be provided.	0.15	3
8. Multi-analyte or multi-parameter methods are preferred versus methods using one analyte at a time.	0.62	3
9. The use of energy should be minimized.	0.0	3
10. Reagents obtained from renewable sources should be preferred.	1.0	4
11. Toxic reagents should be eliminated or replaced.	1.0	4
12. Operator's safety should be increased.	0.8	2

Figure S10. AGREE protocol for GXLE.

Analytical Greenness report sheet

27/07/2022 15:06:37

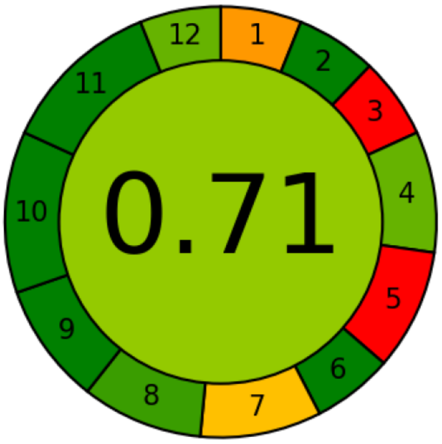

Criteria	Score	Weight
1. Direct analytical techniques should be applied to avoid sample treatment.	0.3	2
2. Minimal sample size and minimal number of samples are goals.	1.0	2
3. If possible, measurements should be performed in situ.	0.0	2
4. Integration of analytical processes and operations saves energy and reduces the use of reagents.	0.8	3
5. Automated and miniaturized methods should be selected.	0.0	3
6. Derivatization should be avoided.	1.0	2
7. Generation of a large volume of analytical waste should be avoided, and proper management of analytical waste should be provided.	0.38	3
8. Multi-analyte or multi-parameter methods are preferred versus methods using one analyte at a time.	0.89	3
9. The use of energy should be minimized.	1.0	3
10. Reagents obtained from renewable sources should be preferred.	1.0	4
11. Toxic reagents should be eliminated or replaced.	1.0	4
12. Operator's safety should be increased.	0.8	2

Figure S11. AGREE protocol for UE.
